# Transcriptomic Analysis in Diabetic Nephropathy of Streptozotocin-Induced Diabetic Rats

**DOI:** 10.3390/ijms12128431

**Published:** 2011-11-29

**Authors:** Consuelo Lomas-Soria, Minerva Ramos-Gómez, Lorenzo Guevara-Olvera, Ramón Guevara-González, Irineo Torres-Pacheco, Marco A. Gallegos-Corona, Rosalía Reynoso-Camacho

**Affiliations:** 1 Research and Graduate Studies in Food Science, School of Chemistry, University of Querétaro, Cerro de las Campanas, S/N, Querétaro, Qro., 76010 Mexico; E-Mails: cons_soria@hotmail.com (C.L.-S.); ramosgomezm@yahoo.com (M.R.-G.); 2 Department of Biochemical Engineering, Technological Institute of Celaya, Av. Tecnológico y Antonio García Cubas s/n, Celaya, Guanajuato, 38010 Mexico; E-Mail: lorenzogo@yahoo.com; 3 Biosystems engineering group, School of Engineering, University of Querétaro, Cerro de las Campanas, S/N, Querétaro, Qro., 76010 Mexico; E-Mails: ramon.guevara@uaq.mx (R.G.-G.); torresirineo@gmail.com (I.T.-P.); 4 Department of Biomedical Research, School of Medicine, University of Querétaro, Clavel 200, Prados de la capilla, Querétaro, Qro., 76017 Mexico; E-Mail: magalle@prodigy.net.mx

**Keywords:** diabetes mellitus, diabetic nephropathy, cDNA libraries, suppression subtractive hybridization

## Abstract

Diabetic nephropathy (DN) is a major complication of diabetes and is caused by an imbalance in the expression of certain genes that activate or inhibit vital cellular functions of kidney. Despite several recent advances, the pathogenesis of DN remains far from clear, suggesting the need to carry out studies identifying molecular aspects, such as gene expression, that could play a key role in the development of DN. There are several techniques to analyze transcriptome in living organisms. In this study, the suppression subtractive hybridization (SSH) method was used to generate up- and down-regulated subtracted cDNA libraries in the kidney of streptozotocin (STZ)-induced diabetic rats. Northern-blot analysis was used to confirm differential expression ratios from the obtained SSH clones to identify genes related to DN. 400 unique SSH clones were randomly chosen from the two subtraction libraries (200 of each) and verified as differentially expressed. According to blast screening and functional annotation, 20.2% and 20.9% of genes were related to metabolism proteins, 9% and 3.6% to transporters and channels, 16% and 6.3% to transcription factors, 19% and 17.2% to hypothetical proteins, and finally 24.1 and 17.2% to unknown genes, from the down- and up-regulated libraries, respectively. The down- and up-regulated cDNA libraries differentially expressed in the kidney of STZ diabetic rats have been successfully constructed and some identified genes could be highly important in DN.

## 1. Introduction

Diabetic nephropathy (DN) is one of the most frequent microvascular complications of diabetes mellitus, and develops in 15–40% of patients with Type 1 and Type 2 diabetes. The course of renal disease might be similar for both types of diabetes. This complication is associated with a considerable increase in morbidity and mortality, being the leading cause of end-stage renal failure (ESRD) [1,2]. The highest incidence of ESRD has been reported for Taiwan, United States, Mexico, China and Japan, and has been found to be related to a higher prevalence of diabetes mellitus. For example, in Jalisco, Mexico, 60% of newly diagnosed ESRD patients had a primary diagnosis of diabetes [3].

Diabetes is characterized by hyperglycemia, an abnormal elevation in the blood glucose level, and it has been associated with oxidative stress [4] and inflammatory processes [5,6], which cause DN. This complication is related to specific morphological and functional renal alterations. They include hyperplasia/hypertrophy of various cell types of the glomeruli and tubules [7], producing changes of renal function, characterized by an increase in urinary albumin excretion in early stages and by proteinuria in advanced stage, which is also considered as a decline in renal function [8].

In recent years, it has been reported that several hemodynamic and metabolic factors are involved in the pathogenesis of DN [9], such as advanced glycation end-products and the aldose reductase/polyol pathway, vasoactive factors such as angiotensin II and endothelin. Also vasodilator factors, such as nitric oxide, are related to DN. In addition, protein kinase C, an intracellular signaling molecule, can be activated by many of these metabolic and hemodynamic factors to stimulate prostaglandin and cytokine production. Several growth factors, such as the transforming growth factor beta, vascular endothelial growth factor and platelet-derived growth factor, are involved in the pathogenesis of diabetic kidney disease [10].

Despite several recent advances, the pathogenesis of DN remains far from clear [11]. Therefore, there is current interest in identifying signaling molecules that play a key role in the management of DN. This could be achieved with the use of newly developed tools like microarray technology, proteomics and transcriptomics.

Several pathologies, such as DN, show a differential gene expression pattern, which could be identified by these techniques. Therefore, subtractive suppression hybridization (SSH) can be used to clone the differentially regulated genes. During SSH-polymerase chain reaction (PCR), the population of mRNAs in one condition is subtracted from the mRNAs from another contrasted condition, thus isolating differentially expressed genes, which can be further characterized [12]. By using the SSH technique, a number of genes from STZ-induced diabetic mouse kidneys were identified, such as Tim44 (translocase of inner mitochondrial membrane-44), RSOR/MIOX (renal specific oxido-reductase/myo-inositol oxygenase), UbA52 and Ras-like GTPase, Rap1b [3], among others. However, the isolation and identification of new biomarkers could serve as therapeutic targets for the amelioration of DN.

One of the most common models used for the evaluation of these alterations is the spontaneously and chemically-[e.g., streptozotocin (STZ)] induced rat model of type 1 diabetes, in addition to rat models of type 2 diabetes. These animals display early renal alterations that share similarities to the early changes seen in human diabetes [10]. Diabetes, induced in Wistar rats by a single intraperitoneal injection of STZ (50 mg/kg), causes: severe destruction of the kidney and glomerular and tubulointerstitial lesions, such as glomerular sclerosis, atrophy, interstitial expansion; and interstitial cellular infiltration, after four weeks [13]. Therefore, in order to detect differential gene expression involved in DN, we used the well-known model of STZ induced diabetes in rats to apply the SSH technique in order to compare between normal and diabetic kidneys.

Therefore, the aim of the present work was to determine a differential transcriptomic profile during DN in diabetic rats by using SSH. Some identified genes are discussed in order to interpret their possible role in DN.

## 2. Results and Discussion

### 2.1. Biochemical Parameters in Healthy and Diabetic Rats

Induced experimental diabetes in animals was performed using a single injection of 50 mg/kg B.W. of STZ. This dose causes partial destruction of pancreatic β-cells responsible for producing and secreting insulin into the body, therefore, rats showed type 1 diabetes. The lack of insulin causes a decrease in the incorporation of glucose into peripheral tissues such as adipose and muscle, thereby increasing blood glucose [14]. In this experiment, STZ-injected rats showed a significant increase in blood glucose with a decrease in insulin levels, producing a diabetic condition (Table 1).

Regarding physiological parameters after induction of diabetes, significant differences in consumption of food and water, growth retardation and urine volume were observed in diabetic animals in comparison to healthy animals. Also, a decreased creatinine clearance (Ccr) was observed in diabetic animals compared with nondiabetic rats (*P* < 0.05). On the other hand, markers of renal damage in urine such as urea, albumin and protein excretion showed an increase in diabetic rats compared with those values obtained in nondiabetic animals (Table 1). Additionally, blood urea nitrogen and serum creatinine levels in diabetic rats indicate progressive renal damage, which is considered to be an indication of altered glomerular filtration rate in DN [15]. Our results are similar to those reported by Akbarzadeh *et al*. [16]. Likewise in this experiment, diabetic rats showed an increase in kidney weight compared to healthy rats (*P* < 0.05). In addition, these results are consistent to those described by Yamabe *et al*. [17], who observed an increase in kidney weight in STZ-induced diabetic male Wistar rats compared to healthy rats. This could be related to kidney hyperplasia caused by inflammation. Overall, these results suggest the presence of abnormalities in the renal function of diabetic animals.

### 2.2. Kidney Histopathology

**Figure 1:**
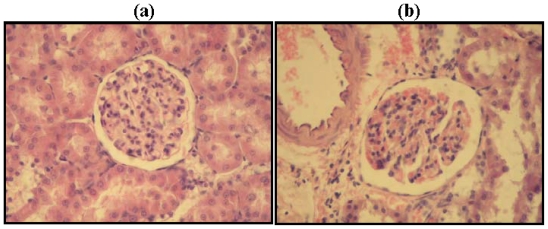
Histological analysis of renal changes in (**a**) healthy and (**b**) diabetic rats. Representative photomicrographs of H-E stained renal cortex.

Besides the biochemical changes in diabetic rats, morphological changes in kidney were carried out to confirm the development of DN. One of the morphological changes associated to DN is mesangial expansion due to increased mesangial matrix deposition, a mild increase in mesangial cellularity, and hypertrophy of mesangial cells [18]. Sections of rat kidney cortices obtained from the two experimental groups were stained with Hematoxylin-Eosin (H-E) and the results are shown in Figure 1.

The kidneys of healthy rats showed normal morphologies of glomeruli and tubules (Figure la). However, kidneys of the diabetic group showed glomerular damage and severe destruction of tubules (Figure lb). These results are similar to those reported by Omotayo *et al.* [19]. It has been observed that structural abnormalities of DN are similar in type 1 and type 2 diabetes and all renal compartments (glomeruli, tubulointerstitium, and renal vasculature) can be affected [20].

In recent years, gene expression profiling related with diabetic nephropathy has been studied in different animal models, such as STZ-induced diabetes in mice and rats, as well as spontaneous rodent models of diabetes. These studies have identified genes involved in the development and progression of this disease, but their precise molecular mechanisms have remained unclear and only a low number of those genes are considered biomarkers [3]. Therefore, it is important to identify new genes involved in this disease that can serve as molecular targets to broaden the development of novel therapeutic strategies.

### 2.3. Differential Expression of Genes

**Figure 2:**
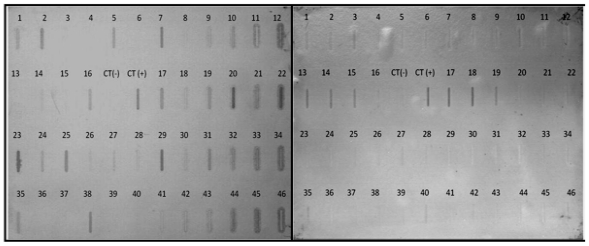
Typical hybridization pattern of cDNA arrays from the library obtained by SSH. Panel left: probe from diabetic kidney rat. Panel right: probe from healthy rat kidney.

By using SSH technique, 400 EST’s were obtained (200 down- and 200 up-regulated) from kidneys of diabetic rats. Northern blot analysis was performed to confirm the differential expression of the EST’s obtained from the cDNA libraries; however only 200 EST’s were transferred to nylon membranes (100 to each library) and hybridized with RNA probes from either diabetic or healthy rats. It was observed that 88 and 72 clones from the up- and down-regulated libraries, respectively, showed a marked differential expression in the presence of DN in STZ-induced rats. Typical expression pattern of cDNA arrangements of 47 clones from the subtractive library obtained is shown in Figure 2.

### 2.4. Analysis of Sequence Homology of EST’s

EST’s, based on their differential expression pattern, were selected and sequenced. The sequences of these clones were compared to those of the database from NCBI by means of the BLAST algorithm, and the results from the found genes are shown in Tables 2 and 3. Based on this, possible functions for the sequenced EST’s were grouped in: (1) Metabolism protein; (2) transporters and channels; (3) hypothetical proteins; (4) transcription factors; and (5) unknown function.

### 2.5. Differential Expression of Selected Transcripts from the Subtractive Library

In the present study, the main objective was to identify some differentially expressed genes involved in DN. As aforementioned, based on hybridization signals, 160 EST’s were selected for sequencing, from which 72 were down- and 88 over-expressed genes (Tables 2 and 3, respectively). BLAST analysis revealed that at least 80% of the genes had been previously reported in the GeneLibrary database. However, 20% of these fragments did not match any gene published in the gen bank, thus raising the possibility of newly identified genes.

### 2.6. Related Function of Transcript Selected from the Subtractive Library

#### 2.6.1. Metabolism Protein

**Figure 3:**
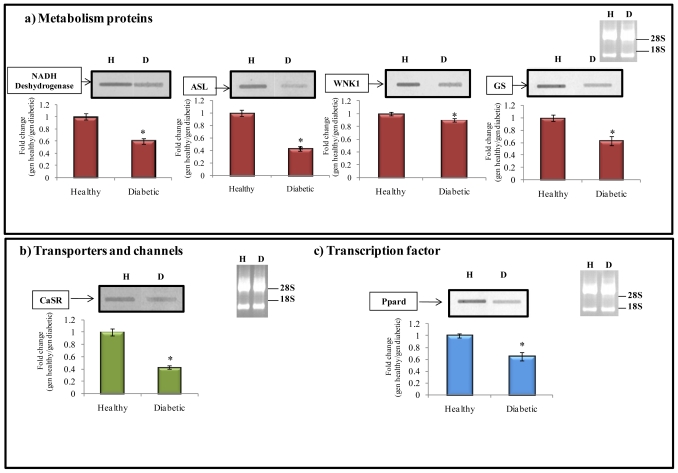
Northern-blot analysis performed in selected genes of the down-regulated library.

Table 2 (Section a) and Figure 3a show the down-regulated genes related to several enzymes that participate in cellular metabolism, e.g., NADH dehydrogenase. This enzyme plays a fundamental role in cellular respiration. This protein is located in the inner mitochondrial membrane and catalyzes the transfer of electrons from NADH to coenzyme Q (CoQ) in the electron transport chain and this activity is related to maintenance of a transmembrane proton gradient used to produce ATP, since mitochondrial energy production is essential for all cellular processes and organ functions. In acute kidney injury (AKI), de-energization of the mitochondria and loss of mitochondrial proteins may lead to irreversible cell injury, limiting restoration of organ function. In primary cultures of renal proximal tubule cells (RPTCs), recovery from oxidant-induced mitochondrial dysfunction was related to an increased expression of the mitochondrial proteins NADH dehydrogenase and ATP synthase β, and elevated mitochondrial respiration rates and ATP levels [21]. In the present study, we found that NADH dehydrogenase was down-regulated in diabetic animals, suggesting that the electron transport chain could be decreased in this condition, thus altering energy production (ATP). Argininosuccinate lyase (ASL) enzyme was also down-regulated. The kidney plays a key role in arginine metabolism. Renal l-arginine (l-Arg) synthesis requires citrulline, made primarily in the small intestine, which is then taken up into the kidney and converted by argininosuccinate synthase (ASS) and argininosuccinate lyase (ASL) into l-Arg. The l-Arg is then released into the circulation via the renal vein, taken up into cells, and utilized in several pathways including nitric oxide (NO) synthesis. Humans and animals with renal disease, show increases in plasma citrulline correlating with decreased renal function [22]. In fact, renal l-Arg production is impaired quite early before any significant structural damage is evident. The fall in arginine production is due to an early loss of ASS and ASL for conversion of citrulline, along with a later reduction in citrulline uptake. In chronic kidney disease (CKD), ASL density was unchanged at 1–2 weeks after injury; however, when adjusted for viable renal mass, there was a 60% reduction in total renal ASL abundance at 1–2 weeks after injury, and no change was observed in more severe CKD [23]. In our experiment, EST’s of ASL were repressed (Table 2, Section a, and Figure 3a) in diabetic kidney compared to healthy rat kidney, suggesting a probable reduction in l-Arg concentration and, therefore, contributing to the resulting NO deficiency.

Also, WNK1 was found to be down-regulated in diabetic conditions (Table 2, Section a and Figure 3a). WNK are protein kinase and WNK1 has multiple alternatively spliced isoforms including long forms expressed in the whole nephron at a low level (l-WNK1) and a kidney specific form (KS-WNK1) distributed in the distal convoluted tubule, the connecting tubule and the cortical collecting duct. KS-WNK1 is 1700 aminoacids in length and lacks aminoacids 1–437 of the long l-WNK1 isoform that are encoded by exon 1–4. L-WNK1 decreases cell surface abundance of renal K^+^ channel ROMK1 (renal outer medullary potassium (K) channel 1) and induces a decrease in K^+^ secretion by the kidney contributing to hyperkalemia. K^+^ secretion by kidney is critical for controlling serum K^+^ levels and overall K^+^ homeostasis. KS-WNK1 is an important physiological antagonist of l-WNK1, and the ratio of l-WNK1 to KS-WNK1 regulates surface abundance of ROMK1 and renal K^+^ excretion [24,25]. Unexpectedly, diabetic kidney showed a down-regulation of WNK1 compared to healthy kidney, thus allowing potassium to exit through transporter protein ROMK, which is usually negatively modulated by WNK1.

**Figure 4:**
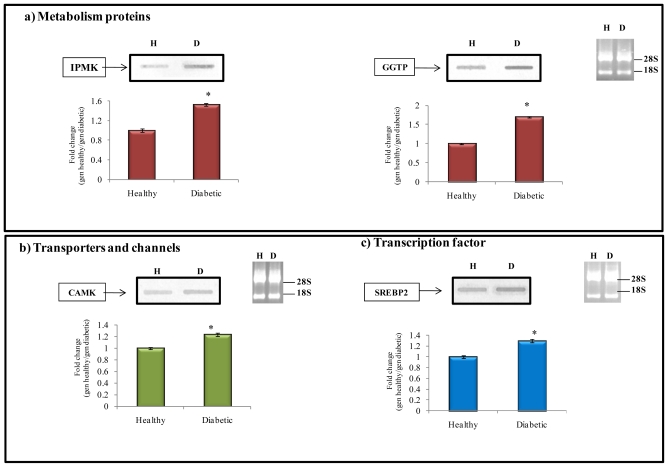
Northern-blot analysis performed in selected genes of the up-regulated library.

Likewise, inositol polyphosphate multikinase (IPMK) is a member of the IP_6_ kinase family of enzymes that generates several inositol phosphates. IPMK also possesses phosphatidylinositol 3-kinase (PI3K) activity, specifically phosphorylating the phosphatidylinositol (4,5)-bisphosphate (PIP_2_) to generate phosphatidylinositol (3,4,5)-trisphosphate (PIP_3_), a second messenger known to promote cellular growth, proliferation, survival, and migration. Genetic deletion of IPMK impairs Akt signaling and diminishes cell growth, consequences that are determined by the PI3K activity of IPMK [26]. In this experiment, over-expression of IPMK (1.4 fold change, Table 3 Section a, and Figure 4a) was found in diabetes condition, and this effect could be related to an increase of cell proliferation of both macrophages and myofibroblasts, likely contributing to the tubulointerstitial infiltration observed in DN.

In addition, gammaglutamyl transpeptidase (GGTP1) was found to be over-expressed in kidney of diabetic rats compared to healthy control group (Table 3 Section a, and Figure 4a). GGTP1 is an enzyme primarily located in the brush border of the proximal convoluted tubules of the kidney. Recent studies have shown that GGTP1 itself plays a marked pro-oxidant role under certain conditions. The cysteinyl-glycine resulting from the GGT-mediated cleavage of GSH reduces ferric iron, thus triggering iron redox cycling reactions, which stimulates the production of hydroxyl radicals and enhances membrane lipid peroxidation that could contribute to a certain degree to morphological changes of tubular cells [27]. In our study, we evaluated the levels of TBARS in kidney of diabetic animals and their content was higher (638 nmol/mg tissue) compared to that of healthy animals (211 nmol/mg tissue); therefore, the results demonstrated the presence of lipid peroxidation in diabetic kidney.

The glutamine synthetase (GS) is an enzyme that converts glutamate and ammonia into glutamine. This enzyme is present in rat kidney and its activity is considered to play a role in limiting the release of the ammonia generated by renal cells into both the urine and renal vein. Therefore, this gene can be related to renal dysfunction. Thus, glutamine synthesis not only detoxifies ammonia, a potentially toxic compound to the nervous system, but also contributes to the regulation of systemic acid-base balance by reducing the urinary excretion of ammonia in the form of ammonium ions [28]. A study demonstrated that the activity of GS decreased by 40% in the outer medulla during metabolic acidosis [29]. In our results, the expression of GS in kidney of diabetic rats was repressed by 40% (Table 2 Section a, and Figure 3a).

#### 2.6.2. Transporter and Channels

Table 2, Section b, is a summary of down-regulated genes encoding transporters and channels in kidney under diabetic conditions. In this regard, the extracellular calcium-sensing receptor (CaSR) gene is shown in Table 2, Section b, and Figure 3b. In the kidney, the CaSR has several different actions, leading to enhanced reabsorption of sodium chloride and increased calcium excretion in the renal tubules [30]. In the nephrectomy rat model, CaSR mRNA expression and CaSR protein levels were reduced by 35 and 38%, respectively, compared to those observed in control animals. The results suggest that renal CaSR expression is reduced in chronic renal insufficiency and this may play a role in disordered mineral ion including hypocalciuria [31]. These results are in accordance with those found in our study.

Regarding the up-regulated genes, the calcium- and calmodulin- dependent protein kinase (CamK) gen was over-expressed (1.2 fold change) in kidneys of STZ-induced diabetic rats (Table 3, Section b, and Figure 4b).

Free cytosolic calcium level plays a crucial role in many important cellular processes, such as gene transcription, cell proliferation and differentiation, and apoptosis. Calcium ions are also known to regulate several enzymes and to interact with a large number of other calcium-binding proteins that participate in several cellular signaling pathways [32].

The Multifunctional CamK is a ubiquitous enzyme that is present in all cell types thus far examined. This enzyme is so named because of its requirement for calcium-bound calmodulin for activation and its ability to phosphorylate and alter the function of a variety of substrates. In mesangial cells, Src protein tyrosine kinases act downstream of CamK in a signaling pathway in which Ca^2+^_i_ induces the *c-fos* promoter and increases DNA synthesis, resulting in hypertrophy, hyperplasia, and/or expansion of the mesangial matrix [33].

#### 2.6.3. Transcription Factors

In the down-regulated library, the transcription factor peroxisome proliferator-activated receptor delta (PPARδ or PPARD) was also identified (Table 2, Section c, and Figure 3c). PPARδ is a ligand-activated transcription factor that belongs to the nuclear hormone receptor family and plays a key role in cell survival [34,35]. In comparison with PPARα and PPARγ, PPARδ is ubiquitously expressed in all nephron segments within the kidney; in addition, this is the predominant isotype in the proximal straight tubule [36]. Because of this high expression, PPARδ would contribute to survival of proximal tubular cells in ischemic acute renal failure [37]. However, with more sustained ischemia/reperfusion, epithelial cells of the proximal tubule undergo necrotic or apoptotic cell death, thus contributing in part to the inflammatory response after renal ischemia/reperfusion. Letavernier *et al*. [37] found that the PPARδ ligands could directly trigger inflammation in endothelial cells. Also, PPARδ ligands have shown to inhibit the expression of proinflammatory cytokines. Therefore, the repressed expression (0.6 fold change) of PPARδ found in our experiment (Figure 3c) could be related with renal dysfunction, since PPARδ exerts a strong protection against renal failure.

On the other hand, several up-regulated transcription factors were identified (Table 3, Section c, and Figure 4c), such as the sterol regulatory element-binding protein-2 (SREBP2). SREBP2 is a member of the sterol regulatory element binding proteins (SREBPs) involved in the regulation of lipid homeostasis. SREBP2 is a transcription factor regarded as the main regulator of cholesterol homeostasis and an increased level of SREBP-2 could be responsible for hypercholesterolemia generally observed in experimental chronic renal failure [38].

It has been shown that an increased renal expression of SREBP1 and SREBP2 results in an excessive accumulation of triglycerides and cholesterol in renal structures, and this lipid accumulation is associated with glomerulosclerosis and proteinuria [39]. In this study, SREPB2 was over-expressed (1.3 fold change) in the kidney of diabetic rats compared to healthy rats (Figure 4c) [40].

#### 2.6.4. Unknown Genes

**Figure 5:**
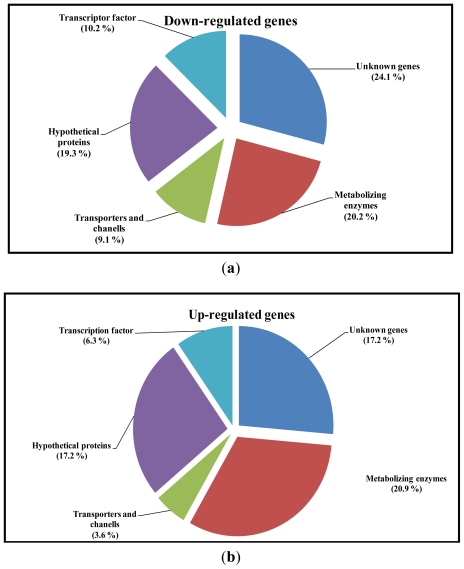
Renal genes isolated by subtractive suppressive hybridization from (**a**) down-and (**b**) up-regulated libraries of STZ-induced rats.

From the library of down-regulated genes, 25 EST’s with unknown functions were found with a repression ratio of between 0.413 and 0.921 times, compared to those of healthy rats. On the other hand, 19 EST’s with unknown functions were obtained in the library of up-regulated genes, with an over-expression ratio of between 1.3 and 2.5 times, compared to those of the healthy group. These represent 24.1 and 17.2% of the total EST’s from down- and up-regulated libraries (Figure 5a,b, respectively).

The overall results are summarized in Figure 5, where it shows the percentage of EST’s found in the down- and up-regulated libraries, based on their possible gene functions. For down-regulated genes: (1) Metabolizing enzymes 20.2%; (2) transports and channels 9.1%; (3) hypothetical proteins 19.3%; (4) transcription factors 10.2%; and (5) unknown function 24.1%. Likewise, for up-regulated genes: (1) Metabolizing enzymes 20.9%; (2) transports and channels 3.6%; (3) hypothetical proteins 17.2%; (4) transcription factors 6.3%; and (5) unknown function 17.2%.

In this study, it was possible to identify genes during the early phases of diabetic nephropathy, such as IPMK and WNK1, which have been previously reported in kidney but had not been associated with the development of this disease.

## 3. Experimental Section

### 3.1. Induction of Diabetes and Experimental Protocol

Animal experiments were conducted in accordance with ethical guidelines of the University of Queretaro. Male Wistar rats (weighing 250–300 g) were obtained from Rismart S.A., Mexico. The animals were housed at 20–25 °C with a 12-h light-dark cycle, through the experimental period. Animals were allowed free access to water and a base diet (Zeigler rat chow) containing: protein, 18%; fat, 4%; carbohydrates, 54%; fiber, 5%; and sufficient minerals and vitamins to maintain the health of the rats. Diabetes was induced by a single intraperitoneal injection of freshly prepared STZ (Sigma Chemical Company, USA, 50 mg/kg body weight; dissolved in a citrate buffer, 0.01 M, pH 4.5) after overnight food deprivation. Nondiabetic rats received the same volume of vehicle (citrate buffer). After 1 week, rats with blood glucose ≥250 mg/dL were considered diabetic for this experiment. Both nondiabetic (group 1) and diabetic (group 2) groups included eight rats each one and were fed with base diet. Blood samples were taken from tail vein and the glucose concentration was measured by a glucose oxidase assay (Glucose Accutrend, Roche, Germany) and measured by a reflective glucometer (Accutrend, GC). One day before sacrifice, animals were housed in metabolic cages to obtain urine for determination of biochemical parameters related to DN. At week four, animals were anaesthetized and blood samples were withdrawn by cardiac puncture and the serum was immediately separated from the blood samples by centrifugation and stored at −80 °C until analysis. Kidneys were immediately removed, thoroughly washed with cold-ice saline phosphate buffer, and weighed. A piece of kidney was stored in formaldehyde solution and another part was frozen in liquid nitrogen and stored at −80 °C until analysis.

### 3.2. Assays of Serum, Urine and Kidney Tissue Samples

DN was assessed biochemically by determining urine volume, urinary urea, albumin, protein and also blood urea. Likewise, blood and urine creatinine levels were used for quantification of creatinine clearance (Ccr). All these parameters were measured using commercial kits (Randox Laboratories Ltd., UK). Creatinine clearance (C_Cr_) was calculated on the basis of urinary Cr, serum Cr, urine volume, and body weight using the following equation: C_Cr_ (mL/min) = [urinary Cr (mg/dL) × urine volume (mL)/serum Cr (mg/dL) × 1440 (min)]. Also, kidney/body weight ratio was calculated. Data were expressed as relative kidney weight (percentage of body weight). Serum insulin levels were determined under fasting conditions using a commercial kit (Millipore, USA) according to the manufacturer’s instructions.

### 3.3. Kidney Histopathology

Renal tissues were fixed in 4% paraformaldehyde solution and embedded in paraffin. Sections were cut at 7 μm with a microtome and deparaffined with xylene. The sections were stained with Hematoxylin-Eosin (H-E) and observed under a light microscope at magnifications of 200×.

### 3.4. RNA Isolation

Total RNA of diabetic and normal rats was obtained by RNEASY protocol (QIAGEN, Hilden, Germany). Integrity and size of RNA was analyzed by means of electrophoresis in agarose gel with formaldehyde. RNA quantification and assessment of purity were measured spectrophotometrically by the absorbance ratio (260/280 nm).

### 3.5. PCR-Selected cDNA Subtraction

1 μg of total RNA of each condition (healthy and diabetic) was used as template for synthesizing the first cDNA chain with Superscript II reverse transcriptase (Life Technologies, Rockville, MD, USA) and the protocol of SMART™ cDNA Synthesis, following the instructions of the supplier (Clontech, Palo Alto, CA, USA). The cDNA was amplified by long distance PCR (LD-PCR) with 15, 18, 21, 24, and 27 parallel cycles to compare and guarantee an optimal and suitable amount of PCR products for the construction of the cDNA subtractive library [12]. CROMA-SPIN^TM^ columns (Clontech, Palo Alto, CA, USA) were used to guarantee highly pure cDNA.

Subtractive hybridization by suppression (SSH) was carried out using the PCR-Select™ cDNA subtraction kit (Clontech, Palo Alto, CA, USA). Tester and driver cDNAs were digested using *Rsa*I to yield blunt ends. In order to obtain up-regulated genes in kidney of diabetic rats, the mRNA of these rats was employed as the “tester” and kidney mRNA of healthy rats as the “driver”. On the other hand, to obtain down-regulated genes from kidney of diabetic rats, kidney mRNA of diabetic rats was employed as the “driver” and kidney mRNA of healthy rats as the “tester”. The tester cDNA fragments were proportionally divided into two aliquots and each one ligated in separated reactions with adapter 1 and adapter 2, resulting in two tester cDNA populations. A small amount of each tester population was mixed with an excess of driver population (5 μg), subsequently heat-denatured, and allowed to anneal for 8 h at 68 °C. The two samples from the first hybridization were combined and annealed overnight at 68 °C with additional fresh-denatured driver cDNA. A primary PCR was conducted to amplify those cDNAs that represented differentially expressed genes. A second PCR was carried out using nested primers 1 and 2 R (Clontech, Palo Alto, CA, USA) to reduce the level of unspecific amplification and for the preparation of Northern blot probes. The products of the secondary PCR amplification were monitored by agarose gel electrophoresis, and the fragments larger than 250 bp were cut out of the agarose gel using a sterile scalpel and purified by using a QIAEXII protocol (QIAGEN, Hilden, Germany).

### 3.6. Cloning, Screening, and cDNA Sequencing Subtracted

The differential PCR fragments were ligated into the PCR 4-TOPO cloning vector, according to the manufacturer’s instructions (Invitrogen, Carlsbad, CA, USA). 2 μL of these ligation reactions was used to transform chemically competent *Escherichia coli* TOP 10 cells (Invitrogen, Carlsbad, CA, USA). The transformed cells were planted on Luria-Bertani (LB) agar containing kanamycin, and the white colonies were selected. Individual *E. coli* colonies were inoculated into LB media containing kanamycin/ampicillin and shaken overnight at 37 °C. Inserts were screened by restriction analysis using *Eco*RI (Invitrogen, Carlsbad, CA, USA) and agarose gel electrophoresis. Each selected colony of the library was kept in cryogenic vials with 500 μL of each culture and 500 μL of 100% sterilized glycerol. The collection of colonies (library of differentially expressed genes) was kept at −80 °C. The products were sequenced and subject to homology search by using BLAST program at the web site of National Center of Biotechnology Information [41].

### 3.7. Northern Blot Analysis

To confirm the expression status of the genes isolated by SSH, Northern blot analysis was performed as previously described [42]. Briefly, the mRNA was isolated from kidneys of diabetic and healthy rats as described above. Of the isolated mRNA, 2 μg were spotted into positive-charged nylon membranes (Hybond^TM^-N^+^ Amersham Bioscience, UK Limited) using the slot manifold (Amersham Bioscience). Probes were created from the previously isolated SSH fragments by incorporating fluorescein-11-dUTP (Gene Images CDP-star random prime labeling module) according to the manufacturer’s instructions (Amersham Pharmacia Biotech Inc. Piscataway, NJ, USA). After ultraviolet (UV) cross-linking the RNA to the membranes (UV stratalinker 2400, stratagem Co., La Jolla, CA, USA), the blots were hybridized, washed, and detected whit the Gene Images CDP-star detection module (Amersham Pharmacia Biotech Inc. Piscataway, NJ, USA) using anti-fluorescein alkaline phosphatase conjugated and CDP-star detection reagent (Amersham Pharmacia Biotech Inc. Piscataway, NJ, USA) and followed by 3 h of exposure.

### 3.8. Statistical Analysis of Expression Data

The *T*-student analysis was performed to evaluate the significant differences (*P* ≤ 0.05) in the expression levels of the diabetic kidney against the healthy kidney (JMP 5.0.1., software). The differential expression analysis consisted of the comparison of band intensity for each gene. The confirmation of the results was validated by Northern blot analysis. The normalization was based on the ratio between the gene of each diabetic control absolute values followed by the ratio of healthy control relative value expressed as fold of change in Tables 2 and 3. The cut off value to assign genes that increase/decrease expression was based on the relative value 1.0 produced by the normalization ratios.

## 4. Conclusions

400 clones were obtained to constitute the up- and down-regulated differentially expressed cDNA libraries from rat kidneys and 200 EST’s were randomly selected and sequenced in order to create a transcriptomic profile. Although several EST’s showed homology with genes whose function could suggest a possible role in the pathogenesis of DN, functional genomics studies need to be performed in order to prove their particular roles in DN.

## Figures and Tables

**Table 1:** Biochemical parameters of healthy and streptozotocin (STZ)-diabetic rats.

Parameters	Healthy control	Diabetic control
**Physiological:**		
Body weight (g)	379.7 ± 12.2 a	248.6 ± 8.3 b
Kidney weight (g/100 g BW)	0.60 ± 0.01 b	0.90 ± 0.02 a
Food intake (g/rat/day)	28.5 ± 0.6 b	56.7 ± 1.9 a
Water intake (mL/rat/day)	40.6 ± 3.6 b	385.8 ± 9.6 a
Urine output (mL/rat/day)	22.4 ± 2.9 b	43.6 ± 3.2 a
Creatinine clearance (mL/min)	1.63 ± 0.04 a	0.78 ± 0.03 b
**Urine (mg/24h):**		
Albumin	1.3 ± 0.2 b	2.6 ± 0.4 a
Protein	4.3 ± 0.3 b	7.2 ± 1.2 a
Urea	5.1 ± 0.6 b	30.4 ± 3.2 a
**Serum:**		
Fasting glucose (mg/dL)	52.0 ± 2.0 b	356 ± 29.1 a
Insulin (μU/mL)	19.1 ± 3.4 a	9.9 ± 1.0 b
Urea (mg/dL)	23.7 ± 1.8 b	80.1 ± 4.9 a

Values are presented as means ± SEM (*n* = 8).

a, b Mean values within a row with unlike superscript letter were significantly different (*P* < 0.05; by the *t*-student test).

**Table 2:** Down-regulated genes isolated by subtractive suppressive hybridization from the kidneys of STZ-induced rats after four weeks.

Function	Accession No.	Blast result	pb	Fold change
**(a) Metabolism proteins**	NM_172034	Farnesyltransferase beta subunit (Fntb)	281	0.742
	AA874879.1	NADH dehydrogenase	388	0.623
	NM_017034	Threonine-protein kinase (PIM-1)	675	0.502
	NM_021577	Argininosuccinate lyase (ASL)	626	0.517
	NM_053774.2	Ubiquitin-specific peptidase 2 (USP2)	251	0.676
	NM_022243.1	Hydroxyisobutyrate dehydrogenase (Hibadh)	368	0.954
	NM_022933.2	Helicase DNA binding protein 8 (Chd8)	202	0.922
	NM_031795.2	UDP glucose ceramide glycosyltransferase (Ugcg)	346	0.564
	AF227741.1	Serine/threonine-protein kinase (WNK1)	423	0.870
	NM_024160.1	Cytochrome b-245, alpha polypeptide (Cyba)	663	0.792
	NM_001009668.1	Electron-transfer-flavoprotein, alpha polypeptide (Etfa)	373	0.506
	M29579.1	Glutamine-synthetase (GS)	372	0.640
	XM_221132.5	Ubiquitin-conjugating enzyme E2O (Ube2o)	226	0.858

**(b) Transporters and channels**	NM_052983.2	Solute carrier family 5 (sodium iodide symporter), member 5 (Slc5a5)	280	0.906
	NM_016996.1	Calcium-sensing receptor (Casr)	417	0.413
	D50497.1	CIC-type chloride channel (ClC-5)	242	0.786

**(c) Transcription factors**	NM_001191711	DEAD box polypeptide 20 (Ddx20)	519	0.793
	NM_013141.2	Peroxisome proliferator-activated receptor delta (Ppard)	580	0.631

**Table 3:** Up-regulated genes isolated by subtractive suppressive hybridization from the kidneys of STZ-induced rats after four weeks.

Function	Accession No.	Blast result	pb	Fold change
**(a) Metabolism proteins**	NM_001009637.1	Leucyl-tRNA synthetase (Lars)	511	1.011
	NM_001039346.1	Zinc finger, DHHC-type containing 16 (Zdhhc16)	250	1.360
	NM_001159739.1	Glutathione-*S*-transferase (Gsta5)	237	1.133
	NM_032080.1	Glycogen synthase kinase 3 beta (Gsk3b)	302	1.143
	NM_001007620.1	Pyruvate dehydrogenase beta (Pdhb)	200	1.697
	NM_134417.1	Inositol polyphosphate multikinase (Ipmk)	295	1.473
	NM_001004214.1	NAD(P)H dehydrogenase, quinone 2 (Nqo2)	596	1.010
	NM_ NP_446292.2	Gamma-glutamyltranspeptidase 1 (Ggtp1)	636	1.755
	NM_001007235.1	Inositol 1,4,5-triphosphate receptor, type 1 (Itpr1)	504	1.093
	NM_012570.1	Glutamate dehydrogenase 1 (Glud1)	327	1.103
	NM_001004252.1	Phenylalanyl-tRNA synthetase, beta subunit (Farsb)	301	1.012
	NM_017072.1	Carbamoyl-phosphate synthase subunit 1 (Cps1)	591	1.38

**(b) Transporters and channels**	NM_173338.1	Solute carrier organic anion transporter family (SIco6c1)	414	1.777
	NM_134468.1	Calcium-and calmodulindependent protein kinase type 1 (Camk1)	256	1.226
	NM_012517.2	Calcium channel, voltage-dependent, L type, alpha 1C subunit (Cacna1c)	200	1.379

**(c) Transcription factors**	NM_001127373.1	ADNP homeobox 2 (Adnp2)	380	1.967
	NM_031346.1	ROD1 regulator of differentiation 1 (S. pombe)(Rod1)	204	1.963
	NM_001033694.1	Sterol regulatory element binding transcription factor 2 (Srebf2)	766	1.331
